# Infection-Induced Resistance to Experimental Cerebral Malaria Is Dependent Upon Secreted Antibody-Mediated Inhibition of Pathogenic CD8^+^ T Cell Responses

**DOI:** 10.3389/fimmu.2019.00248

**Published:** 2019-02-19

**Authors:** Tovah N. Shaw, Colette A. Inkson, Ana Villegas-Mendez, David J. Pattinson, Patrick Strangward, Kathryn J. Else, Simon J. Draper, Leo A. H. Zeef, Kevin N. Couper

**Affiliations:** ^1^Faculty of Biology, Medicine and Health, The Lydia Becker Institute of Immunology and Inflammation, University of Manchester, Manchester, United Kingdom; ^2^Manchester Collaborative Centre for Inflammation Research, The Lydia Becker Institute of Immunology and Inflammation, University of Manchester, Manchester, United Kingdom; ^3^The Jenner Institute, University of Oxford, Oxford, United Kingdom; ^4^Faculty of Biology, Medicine and Health, University of Manchester, Manchester, United Kingdom

**Keywords:** cerebral malaria, T cells, B cells, spleen, brain, antibody

## Abstract

Cerebral malaria (CM) is one of the most severe complications of *Plasmodium falciparum* infection. There is evidence that repeated parasite exposure promotes resistance against CM. However, the immunological basis of this infection-induced resistance remains poorly understood. Here, utilizing the *Plasmodium berghei* ANKA (PbA) model of experimental cerebral malaria (ECM), we show that three rounds of infection and drug-cure protects against the development of ECM during a subsequent fourth (4X) infection. Exposure-induced resistance was associated with specific suppression of CD8^+^ T cell activation and CTL-related pathways, which corresponded with the development of heterogeneous atypical B cell populations as well as the gradual infection-induced generation and maintenance of high levels of anti-parasite IgG. Mechanistically, transfer of high-titer anti-parasite IgG did not protect 1X infected mice against ECM and depletion of atypical and regulatory B cells during 4X infection failed to abrogate infection-induced resistance to ECM. However, IgMi mice that were unable to produce secreted antibody, or undergo class switching, during the repeated rounds of infection failed to develop resistance against ECM. The failure of infection-induced protection in IgMi mice was associated with impaired development of atypical B cell populations and the inability to suppress pathogenic CD8^+^ T cell responses. Our results, therefore, suggest the importance of anti-parasite antibody responses, gradually acquired, and maintained through repeated *Plasmodium* infections, for modulating the B cell compartment and eventually suppressing memory CD8^+^ T cell reactivation to establish infection-induced resistance to ECM.

## Introduction

Malaria remains one of the most prevalent and severe diseases in the world, responsible for 445,000 deaths, principally in Sub Saharan Africa, in 2016 (1). The majority of malarial morbidity and mortality are attributable to a small number of distinct but frequently overlapping complications, of which, cerebral malaria is one of the most severe (2, 3). In endemic regions, children under 5 years of age are disproportionally susceptible to cerebral malaria whereas older children and adults, despite often harboring very high parasite burdens, rarely develop severe disease (4).

It is believed that the age-associated protection from cerebral malaria in endemic regions is driven by repeated parasite-exposure, and resultant reprogramming of anti-*Plasmodium* immunity, rather than natural evolution of immune components in the maturing host immune system (5–9). Indeed, adults remain susceptible to severe malarial disease in non-endemic malarial areas and in regions of unstable transmission (4). Nevertheless, the precise number of infectious episodes necessary to provide immunity to severe malaria has yet to be definitively defined (10). Moreover, despite substantial research, the nature, and identity of the immune responses that develop following natural repeated exposure to prevent cerebral malaria are also poorly understood (5–9).

Anti-parasite antibodies, Foxp3^+^ regulatory T cells, IL-10 secreting T cells, and tolerance to malaria toxins, such as GPI and hemozoin, have all been postulated to play important roles in the establishment of immune balance during malaria; however, how these responses develop and their relative contribution to infection-induced protection against severe disease, is mostly unknown (5–9, 11, 12). There is increasing evidence that atypical B cell populations characterized by expression of various markers including CD11c and T-bet develop in response to chronic and repetitious *Plasmodium* exposure (13, 14). Nevertheless, the function of atypical B cells and their influence in regulating resistance to severe malarial complications remain unclear (13–19). Indeed, whilst atypical memory B cells have been shown to be superior active sources of anti-parasite antibody than traditional memory B cells (15), in other studies they have been shown to exhibit restricted activation and impaired capacity to differentiate into antibody or cytokine producing cells following re-stimulation (13). Atypical memory B also express high levels of regulatory receptors and may exert important immunoregulatory functions in suppressing inflammation during malaria, contributing to anti-disease immunity (14, 17–19).

In this study we have utilized the *Plasmodium berghei* ANKA (PbA) murine model of experimental cerebral malaria (ECM) to examine the immunological basis of exposure-induced resistance against malarial cerebral pathology. To date, the ECM model (20) has been significantly underused in the study of immune mechanisms that provide infection-induced protection against severe malaria disease. Indeed, the physiological immune pathways developed following repeated PbA infection that prevent ECM remain unknown. Here we demonstrate that three rounds of infection-drug cure are required to promote robust resistance to ECM during subsequent fourth (4X) infection. We show that infection-induced resistance to ECM is associated with the significant expansion of atypical B cell populations and repression of memory CD8^+^ T cell reactivation, and that this protection is abrogated in mice unable to produce secreted antibody, or undergo class switching. Protection, however, cannot be recapitulated in primary infected mice following passive transfer of plasma from resistant repeatedly infected mice. Moreover, protection is intact in repeatedly infected mice in which atypical and regulatory B cells are acutely depleted. The results in this study provide new evidence for the importance of antibody in mediating protection against cerebral malaria and suggest antibody is required throughout and post-repeated infections to orchestrate gradual modulations in immune responses that ultimately underpin protection.

## Materials and Methods

### Mice and Infections

6–8 week old C57BL/6 mice were purchased from Charles River. IgMi mice (21, 22) and littermate WT mice were bred at the University of Manchester. All mice were maintained in individually ventilated cages at the University of Manchester. BALB/c mice were used for production of *P. berghei* parasite lysate at the University of Oxford. Cryopreserved GFP-expressing *P. berghei* ANKA parasites of clone 15cy1 (23) were thawed and passaged through mice before being used to infect experimental mice via intravenous (i.v.) injection of 1 × 10^4^ parasitized red blood cells (pRBCs) in the tail vein. Infected mice were monitored for neurological symptoms (paralysis, ataxia, convulsions, and coma occurring between day 6 and 10 post-infection). Parasitemia was measured from day 3 post-infection (p.i.) by examination of Giemsa-stained thin blood. Drug cure was achieved on specified days by six, daily, intraperitoneal (i.p.) injections of 30 mg/kg chloroquine combined with 30 mg/kg artesunate in PBS. In some experiments mice were administered on indicated days of 4X infection, via intraperitoneal (i.p.) injection, 300 μg anti-IL-10R (1B1.3A), 250 μg anti-CD20mAb (5D2), or 250 μg anti-Ragweed (control) mAb (Abs from Bioxcell or Genentech, Inc.). In some experiments mice were administered on indicated days of 1X infection, via i.p. injection, 500 μl of heat inactivated plasma (heated at 56°C for 30 min to destroy cytokines and complement), obtained from 4X infected mice (day 7/8 of infection) or from age matched naïve mice. ECM development was assessed using a well-established grading system (24): 1 = no signs; 2 = ruffled fur/and or abnormal posture; 3 = lethargy; 4 = reduced responsiveness to stimulation and/or ataxia and/or respiratory distress/hyperventilation; 5 = prostration and/or paralysis and/or convulsions. Stages 2–3 were classified as prodromal signs of ECM and stages 4–5 were classified as late stage ECM. All animals were euthanized by rising concentration of CO_2_ when observed at stage 4 or 5.

### RNA Isolation

Spleen sections were isolated from mice prior to whole-body perfusion of PBS. Brains were isolated from mice following intracardial whole-body perfusion of PBS. Tissue was snap frozen in liquid nitrogen and stored at −80°C until use. RNA isolation from spleen sections and whole brains was performed by homogenizing brains in Trizol and using lipid tissue RNA easy kits according to the manufacturer's instructions (RNeasy Lipid Tissue Mini Kit, Qiagen). Isolated RNA was DNase treated to remove genomic DNA prior to QC analysis and use in microarray or NanoString analysis.

### Microarray and Gene Expression Analysis

The global gene expression profiles of brains from uninfected, 1X infected, post-3X infected mice and 4X infected mice were probed using the Affymetrix GeneChip® Mouse Genome 430 2.0 microarray containing 34,000 genes. Technical quality control and outlier analysis was performed with dChip (V2005) [www.dchip.org: (25)] using the default settings. Background correction, quantile normalization, and gene expression analysis were performed using RMA in Bioconductor (26). To establish relationships and compare variability between samples, principal components analysis (PCA) was used as this method reduces the effective dimensionality of complex gene-expression space without significant loss of information (27). PCA was performed with Partek Genomics Solution (version 6.5, Copyright 2010, Partek Inc., St. Charles, MO, USA). Differential expression analysis was performed using Limma using the functions lmFit and eBayes (28). 1,957 probe-sets were identified by pairwise comparisons (fold change < or > 1.5 and qvalue < 0.05) between 1X infected vs. uninfected and/or 4X infected vs. aged uninfected groups. The expression level of each probeset was normalized to the naïve average (in log scale, the naïve average expression was calculated and subtracted from each expression level), and then the standard deviation was normalized to 1 (expression level was divided by the standard deviation). The differentially expressed probesets were ranked by clustering the mean expression levels in each group (expression in each group normalized by z-transformation of the mean in log scale), by k-means clustering into 5 clusters, and then ranked by hierarchical clustering. Gene ontology analysis was performed using DAVID Functional Annotation Bioinformatics Microarray Analysis database.

### Nanostring Analysis

The nCounter Gene Expression assay (Nanostring Technologies, Seattle, USA) was performed according to the manufacturer's instructions. Transcript counts were normalized to the relevant housekeeping genes using the nSolver Analysis Software (vers. 2.5; Nanostring Technologies).

### Flow Cytometry

Spleens were mashed and cell suspensions were generated by homogenizing tissue through a 70 μm cell sieve (BD Biosciences) and subjected to RBC lysis (BD Bioscience). Brains were finely minced and leukocytes isolated using the single-step percoll method (29). Cell pellets were subjected to a RBC lysis step. Absolute cell numbers were determined by microscopy using a haemocytometer and live/dead differentiation was performed using the trypan blue exclusion cell viability assay (Sigma). Isolated leukocytes were surface stained with anti-mouse CD3 (17A2), CD4 (GK1.5), CD8 (53-6.7), CD11c (N418), CD19 (6D5 or 1D3), CD45 (30-F11), CD62L (MEL-14), CD80 (16-10A1), CD138 (281-2), ICOS (15F9 or C398.4A), KLRG1 (2F1), IgD (11-26c.2a), IgM (RMM-1), MHC II (M5/114.15.2), PDCA-1 (927), and PDL-1 (MIH5 or 10F.9G2). Intracellular staining for Foxp3 (FJK-16s), granzyme B (GB11), IFNγ (XMG1.2), Ki67 (SolA15) and Tbet (4B10) was performed, after treatment with Foxp3 fixation/permeabilisation buffer (eBioscience). IFNγ staining was performed following 4 h *in vitro* stimulation of splenocytes with 50 ng/mL PMA / 2.5 ug/mL ionomycin. Dead cells were excluded using LIVE/DEAD® Fixable Blue Dead Cell Stain Kit (Life Technologies). Fluorescence minus one (FMO) controls were used to set gates. Cells were analyzed with a BD LSR II or Fortessa (Becton Dickinson) using BD FACSDiva software (Becton Dickinson). Data were analyzed with FlowJo (Tree Star Inc.). All antibodies were from eBioscience and Biolegend.

### Histology

Brains were processed and stained with Haematoxylin/Eosin (H & E) as described (17). Briefly, brains were isolated from mice perfused with PBS followed by 4% paraformaldehyde (pfa). Brains were stored in 20% sucrose/4% pfa for 24 h at (4°C) before being transferred to 20% sucrose/PBS for a further 24 h (4°C). Coronal sections were cut using a sledge-microtome at a thickness of 30 μm. Sections were mounted and stained with Haematoxylin/Eosin.

### Immunofluorescence

Brain sections were stained with anti-GFP (A-21311, Life Technologies) and anti-CD31 (MEC 13.3, BD Pharmingen) as described (20). Sections were counterstained in DAPI (Sigma-Aldrich) then cover-slipped in ProLong Diamond anti-fade Mountant (Life Technologies).

### ELISA

Serum anti-*P. berghei* merzoite surface protein 1 C-terminal 19 kDa region (*Pb*MSP1_19_) and anti-*P.berghei* ANKA antibody endpoint titers were determined as previously described (30), Briefly, 96 well ELISA plates (Thermo Scientific) were coated with *Pb*MSP1_19_-glutathione S-transferase (GST) fusion protein [0.5 μg/ml] or *P.berghei* ANKA blood-stage parasite lysate, generated as previously described (31) [0.5 μg/ml], respectively, before being incubated overnight at 4°C, blocked with PBS with 1% BSA at 37°C for 1 h, and diluted mouse sera added in duplicate wells. Plates were incubated at 37°C for 2 h before antibodies were detected using alkaline phosphatase conjugated goat anti-mouse total IgG (Sigma-Aldrich) (1:3,000) for 1 h at 37°C and developed with p-nitrophenylphosphate (Sigma-Aldrich) with absorbance readings at taken at OD_405nm_. Anti-GST responses were obtained by running concurrent wells coated with GST protein [0.29 μg/ml] and subtracted from the respective *Pb*MSP1-GST OD_405nm_ readings. Endpoint titers were determined as the calculated dilution at which the OD_405nm_ equaled no sera control wells for anti-parasite, and zero for the GST subtracted anti-*Pb*MSP1_19_ ELISA.

### Quantification of Plasma Cytokine Levels

The concentrations of IL-2, IFN-γ, TNF, and IL-10 in plasma were measured using a ProcartaPlex Mouse Cytokine & Chemokine Panel (26 plex) (eBioscience) on a Luminex® 100/200™ System, following the manufacturer's instructions.

### Statistical Analysis

All statistical analyses were performed using GraphPad PRISM (GraphPad Software, USA). Data were tested for normality using the Shapiro-Wilk normality test. For normally distributed data, comparisons between two groups were made using a Student's *t*-test, with Welch's correction and between multiple groups using a one-way ANOVA with Tukey's test for multiple comparisons. For non-parametric data, comparisons between two groups were made using a Mann-Whitney test and between multiple groups using a Kruskal-Wallis with Dunn's test for multiple comparisons. N-numbers in figure legends refer to the number of biological replicates used to generate the data shown in the figure.

## Results

### Repeated Cycles of Infection and Drug-Cure Protects Mice Against ECM

**Figure 1:**
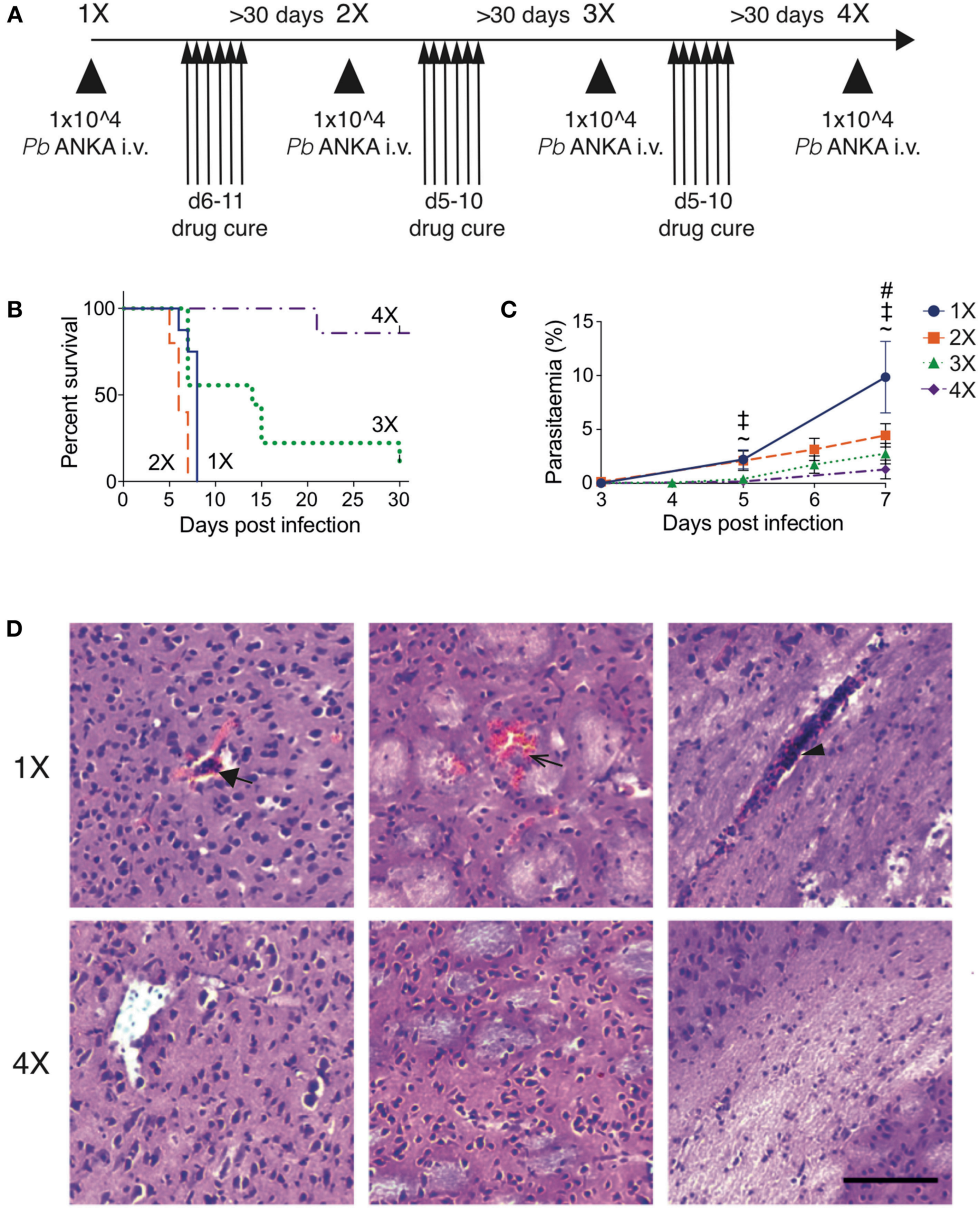
Three rounds of infection-drug cure promote resistance to ECM in susceptible C57BL/6 mice. **(A)** Schematic of the experimental design. C57BL/6 mice were infected with PbA (10^4^ pRBCs i.v.) or left uninfected. Mice were treated (i.p.) with chloroquine and artesunate on stated days post each infection, and re-infections were performed after a minimum interval of 30 days following cessation of drug treatment. **(B)** Kinetics of ECM development shown as percentage of survival of mice. *N* = 7–10 per group, pooled from two independent experiments. **(C)** Peripheral parasitaemia (% of pRBCs) ± SD in different infection groups. *N* = 5–9 per group. Results are pooled from two experiments for the 1X, 2X, and 3X infection and from 3 experiments for the 4X infection. Kruskal-Wallis test with Dunn's multiple comparisons test was used for statistical analysis *p* < 0.05 denoted by #, for 1X v 3X, ‡ for 1X v 4X, and ~ for 2X v 4X. **(D)** Representative H & E stained cortical brain sections demonstrating presence (in 1X infected mice) and absence (in 4X infected mice) of cerebrovascular pRBCs (filled arrow), hemorrhage (unfilled arrow) and leukocyte packed vessels (arrow head) in 1 and 4X infected mice on day 8 post infection. Scale bar 100 μm.

To recapitulate the natural repeated *Plasmodium falciparum* infections experienced by humans in endemic regions (10) we adapted the established PbA model of ECM (Figure 1). C57BL/6 mice were infected with PbA and treated with anti-malarial drugs (artesunate, the front line treatment for severe malaria and chloroquine, as a representative quinolone-containing drug) prior to the development of fulminant ECM (on day 5 or 6, depending upon infection cycle) (experimental schematic in Figure 1A). This cycle of infection-drug cure was repeated up to three times, with a minimum interval of 30 days between cessation of drug treatment and reinfection. Mice gradually acquired resistance to ECM, with mice experiencing a fourth infection (4X infected) being highly resistant to cerebral complications, which typically developed between 6 and 8 days post-infection (Figure 1B). Repeatedly infected mice also gradually acquired a degree of parasite control as 4X infected mice exhibited reduced parasite burdens compared with other infected groups during the early phase of infection (Figure 1C); however, many of the mice subsequently developed transient high level peripheral parasite burdens, which were cleared in 80% of mice by day 30 of infection (Figure S1A and Supplementary Table 1). As expected, very few histopathological features of ECM [as defined in [**20**]], were observed in qualitative analyses of brains from 4X infected mice, in contrast to evidence of pRBC accumulation (left panel), hemorrhage (middle panel) and blocked vessels (right panel) observed in brains from 1X infected mice (Figure 1D and Figure S1B).

This model, therefore, mimics the gradual, infection-induced resistance to cerebral malaria seen in humans, in which resistance to cerebral pathology corresponds with reduced parasite accumulation in the brain and partial, albeit incomplete, control of patent peripheral parasitaemia.

### Repeated Parasite Exposure Regulates Intracerebral CD8^+^ T Cell Activity

**Figure 2:**
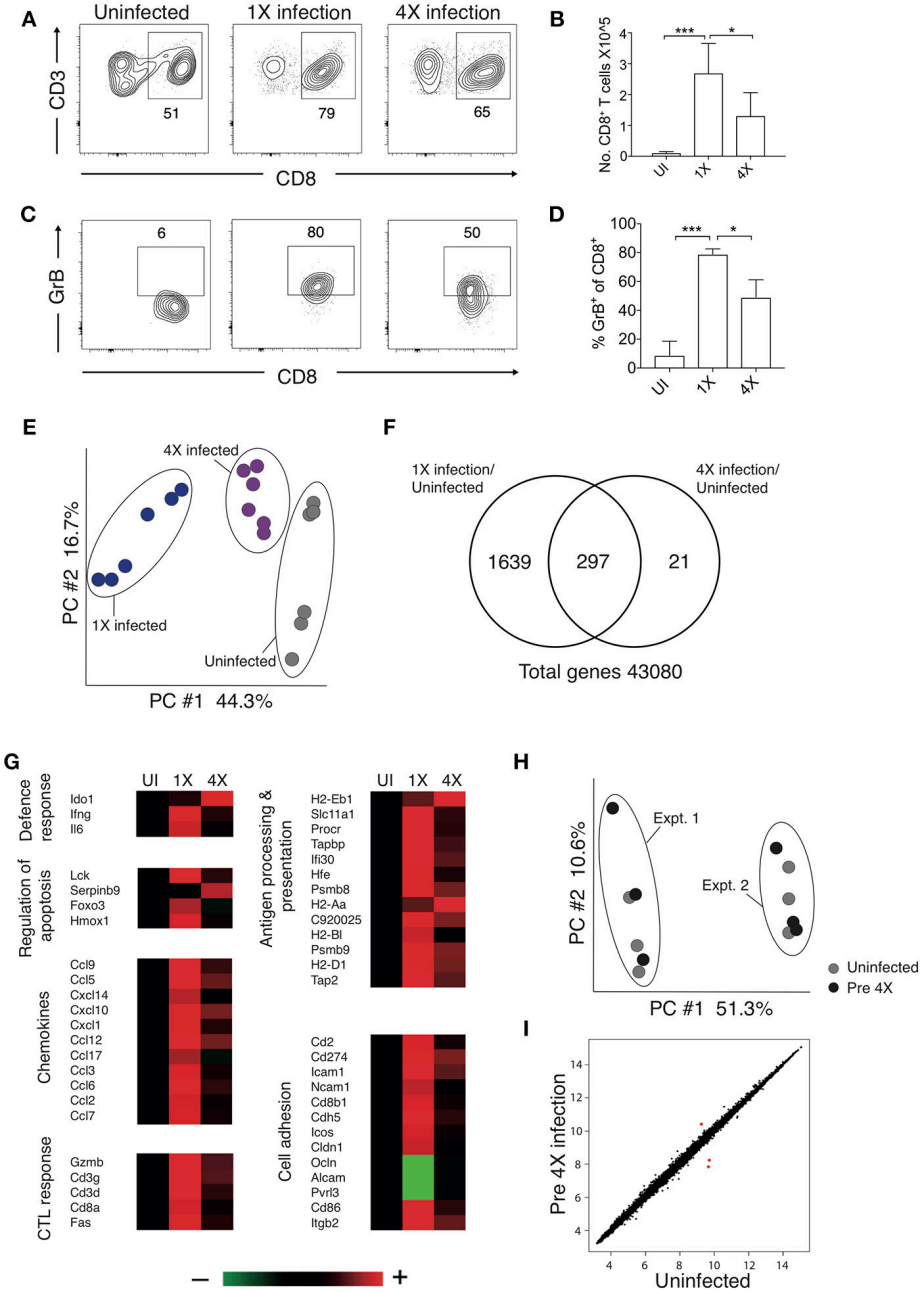
4X PbA infected mice have a distinct whole brain transcriptional signature **(A–D)** Perfused whole brains were removed from 4X infected and age-matched 1X infected C57BL/6 mice on day 8 p.i. (when 1X developed ECM), and age-matched naïve mice. **(A)** Representative flow cytometric plots showing identification of and **(B)** numbers of CD8^+^ T cells in the brain (mean ± S.D.). **(C)** Representative flow cytometric plots showing granzyme B expression by and **(D)** frequencies of granzyme B expressing CD8^+^ T cells in the brain (mean ± S.D.). **(A,C)** Numbers denote the percentage of cells within the gate. **(B,D)** *N* = 4–8 per group, pooled from two independent experiments. Statistical analyses were performed with Kruskal-Wallis test with Dunn's multiple comparisons test or with one-way ANOVA with Tukey's test, depending on normality of data (^*^p ≤ 0.05 and ^***^p ≤ 0.001). **(E–G)** Microarray analysis was performed on perfused whole brains from 4X infected and age-matched 1X infected C57BL/6 mice on day 8 p.i. (when 1X developed ECM), and age-matched naïve mice. **(E)** Principal components analysis of whole-brain transcriptomes. *N* = 6 per group. Results are generated from the pooled array data from brains taken from two independent experiments. **(F)** Venn diagrams defining unique and overlapping genes differentially expressed between 4X infected vs. uninfected mice and 1X infected mice vs. uninfected mice. **(G)** Genes in **(F)** and Supplementary Figure 2 were filtered to identify genes differentially expressed in brains of 1 and 4X infected mice on day 8 of infection. Heat maps showing differentially expressed genes grouped by enriched biological processes identified within DAVID bioinformatics database. Results (*n* = 6 per group) are generated from the pooled array data from brains taken from two independent experiments. **(H,I)** Perfused whole brains were removed from pre-4X infected mice (minimum 30 days post clearance of 3X infection) and age-matched uninfected mice for microarray analysis. **(H)** Principal components analysis of whole-brain transcriptomes (*n* = 6 per group). Results are generated from the pooled array data from brains taken from two independent experiments. **(I)** Scatter plot comparing gene expression between pre-4X infected mice and uninfected mice. Each data point represents the mean expression level of a gene. Results (*n* = 6 per group) are generated from the pooled array data from brains taken from two independent experiments.

Brain migrating CD8^+^ T cells play a major role in the pathogenesis of ECM (32). Thus, we investigated whether infection-induced resistance to ECM was associated with the attenuation of intracerebral CD8^+^ T cell responses. As anticipated, the numbers of CD8^+^ T cells were reduced in the brains of 4X infected mice (day 8: no ECM) compared within brains of 1X infected mice (day 8: late stage ECM) (Figures 2A,B). Moreover, CD8^+^ T cells displayed a less activated phenotype in the brains of 4X infected mice compared with 1X infected mice, as evidenced by significantly lower expression of granzyme B (Figures 2C,D).

To assess the potential intracerebral events that led to reduced CD8^+^ T cell recruitment and CTL functionality in 4X infected mice, we contrasted the transcriptome of whole brains from 1X infected mice, from 4X infected mice, and from uninfected mice. Brains from the three different groups (from day 8 of both 1X and 4X infection) exhibited distinct transcriptional signatures (Figures 2E,F). Genes differentially expressed in brains of 1X infected or 4X infected mice compared with brains from uninfected mice clustered into 5 distinct clusters, with various different biological pathways relating to immune system activation and function being enriched in each cluster (Figure S2, Supplementary Table 2).

Filtering the dataset in Figure S2 (i.e., the genes differentially expressed in brains of 1X or 4X infected mice compared with naïve mice) further, we identified the genes that were also differentially expressed specifically in brains of 4X compared with 1X infected mice (1402 genes in total: Supplementary Table 3). These filtered genes were significantly enriched within immunological processes that included defense response, regulation of apoptosis, chemotaxis, CTL activity, antigen processing and presentation, and cell adhesion (selected genes in the biological processes are presented in Figure 2G and full pathways are presented in Figure S2). In general, the majority of the pro-inflammatory genes (including IL-6 and IFN-γ gene networks) were expressed at higher levels in brains from 1X infected compared with 4X infected mice (Figure 2G and Figures S2, The differences in expression of key genes involved in antigen processing and presentation, chemotaxis and CTL activity was verified by NanoString analysis (Figure S3). Importantly, the brain transcriptome in pre-4X infected mice (i.e., mice that had undergone three rounds of PbA infection and drug cure but did not receive a further infection) was almost identical to that observed in uninfected mice (Figures 2H,I).

Combined, these results show that exposure-induced resistance to ECM is associated with both the reduction in CD8^+^ T cell accumulation and CD8^+^ T cell cytolytic activity within the brain. Moreover, transcriptional differences in the brain are only apparent during active infection, and are not maintained between infections.

#### Exposure-Induced Resistance to ECM Corresponds With Specific Inhibition of Splenic CD8^+^ T Cell Cytolytic Capacity and Not Generalized Suppression of Splenic Effector T Cell Responses

**Figure 3:**
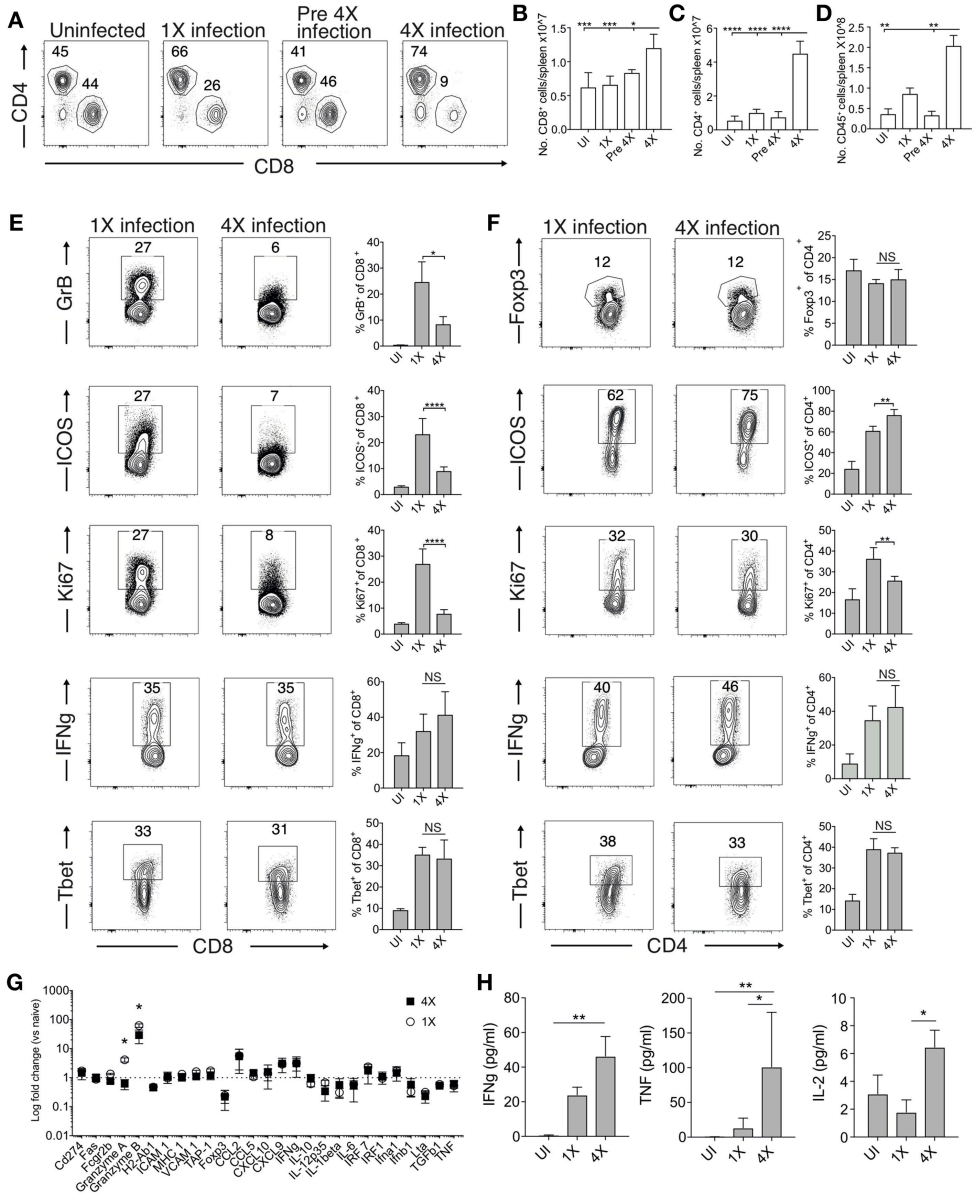
4X infected mice develop significantly altered splenic CD8^+^ and CD4^+^ T cell responses. **(A–H)** Spleens and plasma were removed from 4X infected and age-matched 1X infected C57BL/6 mice on day 8 post-infection (when 1X infected mice developed ECM), from pre-4X infected C57BL/6 mice (minimum 30 days post clearance of 3X infection), and age-matched uninfected mice. **(A)** Representative flow cytometric plots and the frequencies of splenic CD8^+^ T cells and CD4^+^ T cells, gated on live CD45^+^ CD3^+^ cells. Total numbers of splenic **(B)** CD8^+^ T cells, **(C)** CD4^+^ T cells and **(D)** CD45+ cells. **(E,F)** Representative flow cytometric plots and the frequencies of splenic **(E)** CD8^+^ T cells and **(F)** CD4^+^ T cells expressing markers of activation, function and differentiation. **(G)** Nanostring analysis of gene expression in whole spleen tissue from 4X and 1X infected mice (on day 8 of infection), expressed relative to gene expression in age-matched uninfected mice (fold change of 1 defines level of gene expression in uninfected mice). **(H)** Cytokine bead array of plasma cytokine levels in 4X, 1X infected mice and aged matched uninfected C57BL/6 mice. **(A,E,F)** Numbers denote the percentage of cells within the gate. **(B–G)** Results are the mean ± SD of the group, representative of two independent experiments with **(B–D)** *n* = 3–6 per group, **(E,F)** *n* = 3–10 per group and **(G)** *n* = 3 per group. **(H)** *N* = 4–7 per group, pooled from two independent experiments. **(B–F,H)** Statistical analyses were performed with Kruskal-Wallis test with Dunn's multiple comparisons test or with one-way ANOVA with Tukey's test, depending on normality of data (^*^p ≤ 0.05, ^**^p ≤ 0.01, ^***^p ≤ 0.001, ^****^p ≤ 0.0001). **(G)** Statistical analysis by Student's *t*-test, with Welch's correction (^*^p ≤ 0.05).

The absence of maintained transcriptional alterations in the brain between infections suggested that infection-induced protection might be controlled at the site of T cell priming, namely the spleen. We therefore examined splenic T cells responses in 1X and 4X infected mice. The numbers of splenic CD8^+^ and CD4^+^ T cells were significantly increased in 4X infected mice compared with 1X infected mice on day 8 of infection, contributing to splenomegaly in 4X infected mice (Figures 3A–D). T cell numbers were not significantly raised in pre-4X infected mice compared with uninfected or 1X infected mice, showing that expansion of the lymphocyte populations occurred rapidly during the active fourth infection (Figures 3A–C).

Despite their significant expansion, CD8^+^ T cell activation and effector function was significantly attenuated in 4X infected mice compared with 1X infected mice, as shown by reduced expression of granzyme B, ICOS and Ki67 (Figure 3E). Interestingly, however, the frequencies of splenic CD8^+^ T cells expressing T-bet and IFN-γ were similar in 4X and 1X infected mice (Figure 3E). In contrast, the activation and differentiation of splenic CD4^+^ T cells was generally unaltered in 4X infected mice compared with 1X infected mice, with only a minor reduction in Ki67 and upregulation in ICOS expression by CD4^+^ T cells in 4X infected mice (Figure 3F). The frequencies of CD4^+^ T cells expressing Foxp3 was not significantly different in 4X and 1X infected mice, suggesting that altered regulatory T cell development was not the major reason for suppression of CD8^+^ T cell activity and prevention of ECM in 4X infected mice (Figure 3F).

Studying the splenic and systemic immune signature in 4X infected mice compared with 1X infected mice in more detail revealed that the gene expression of granzyme B and granzyme A was significantly reduced in 4X infected mice compared with 1X infected mice (examining gene expression in whole spleen tissue), whereas there was no significant differences in expression of 25 other immune response genes, including antigen presenting molecules (TAP-1, MHC-1), pro-inflammatory chemokines (CXCL9, CXCL10, CCL2, CCL5), and cytokines (TNF, IFN-γ, LT-α) (Figure 3G). Moreover, plasma levels of TNF, IFN-γ and IL-2 were comparable or higher in 4X infected mice than in 1X infected mice (Figure 3H).

Collectively, our results, therefore, show that infection-induced resistance to ECM was associated with the specific reduction in splenic CD8^+^ T cell cytotoxic functions and granzyme expression, rather than non-selective suppression of adaptive T cell expansion and activation, or general dampening of inflammation.

#### Infection-Induced Resistance Is Associated With Generation of Heterogeneous Atypical and Regulatory B Cell Populations and Improved Anti-parasite Ab Responses

**Figure 4:**
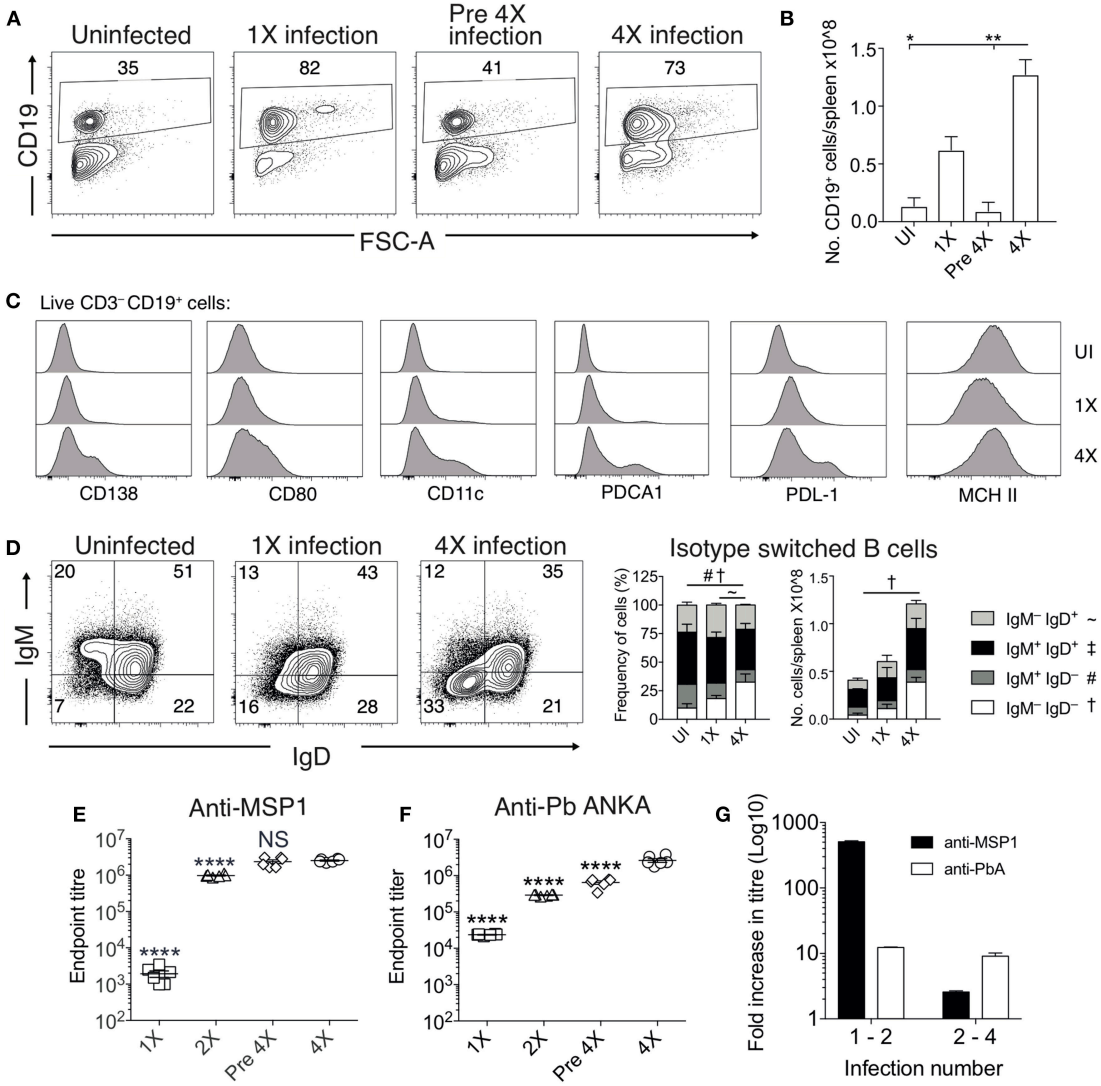
Anti-parasite IgG titres and atypical B cell populations are significantly increased in 4X infected mice. **(A–G)** Spleens and plasma were obtained from 4X infected and age-matched 1X infected C57BL/6 mice on day 8 post-infection (when 1X infected mice developed ECM), from pre 4X infected mice (minimum 30 days post-clearance of 3X infection) and from age-matched uninfected mice. **(A)** Representative flow cytometric plots and the frequencies of splenic CD19^+^ B cells, gated on live CD45^+^ CD3^−^ cells. Numbers denote the percentage of cells within the gate. **(B)** Total numbers of splenic CD19^+^ B cells. **(C)** Expression of CD138, CD80, CD11c, PDCA1, PDL-1, and MHC II by CD19^+^ splenic B cells from uninfected, 1X infected and 4X infected mice. **(D)** Left, Representative flow cytometric plots (gated on live CD45^+^CD3^−^CD19^+^ cells), right, the frequencies and numbers of non-switched (IgM^+^IgD^+^ and IgM^−^IgD^+^) and switched (IgM^+^IgD^−^, and IgM^−^IgD^−^) splenic CD19^+^ B cells. The end point plasma titres of **(E)** anti- PbMSP1_19_ and **(F)** anti-PbA IgG, with background titer from naïve mice subtracted. **(G)** Graphical representation of the fold change in Ab titer between 1X and 2X infected mice and between 2X and 4X infected mice. **(B,D)** *N* = 2–6 per group, representative of two independent experiments (mean ± SD). Statistical analyses were performed with Kruskal-Wallis test with Dunn's multiple comparisons test ^*^p ≤ 0.05, ^**^p ≤ 0.01, and *p* < 0.05 denoted by #, ~, ^†^for indicated groups. **(E,F)** *N* = 6 per group, pooled from two independent experiments (mean ± SD). Statistical analyses were performed by one-way ANOVA with Tukey's multiple comparisons test, shown for 4X infected mice compared to each other group (^****^p ≤ 0.0001).

Chronic and repetitive *P. falciparum* infection is associated with the development of atypical B cell populations, the functions of which are still debated (13–19). As well as being potentially important sources of anti-parasite Ab (15), they may also exert immune regulatory activity, suppressing inflammation (14, 17–19). Consequently, we investigated whether repeated PbA infection led to the formation of atypical B cell populations and how this may contribute to infection-induced resistance to ECM. The numbers of splenic CD19^+^ B cells were significantly increased in the 4X infected mice compared with 1X infected mice, examined on day 8 of infection (Figures 4A,B). Whilst there was a large increase in the frequencies of CD19^+^CD138^+^ (putatively plasmablasts) and CD19^+^CD80^+^ [activated and memory B cells (33)] in 4X infected mice compared with 1X infected mice, we also observed an increase in the frequencies of atypical CD19^+^ cells expressing CD11c [B cells that exert APC activity and may contribute to autoimmunity (13, 34–36)], PDCA-1 [suppressor B cells but which also produce Abs (37)] and PDL-1 [putatively regulatory B cells (38)] in 4X infected mice compared with 1X infected mice (Figure 4C).

The frequencies and numbers of class-switched IgD^−^IgM^−^ B cells were also significantly increased in 4X infected mice compared with 1X infected mice (Figure 4D), which corresponded with significantly higher plasma titers of anti-*Pb*MSP1_19_ and total anti-PbA IgG antibodies in 4X infected mice than 1X infected mice (Figures 4E,F). Of interest, total anti-PbA Ab responses developed much more gradually upon repeated infections than anti- *Pb*MSP1_19_ Ab responses (which largely peaked during 2X infection when mice were still highly susceptible to ECM), (Figures 4E–G). The levels of anti-*Pb*MSP1_19_ IgG were not boosted at all during the fourth infection (when measured on day 8 of infection and compared with levels in pre-4X infected mice), whereas the levels of total PbA-specific IgG were boosted only slightly during the 4th infection (Figures 4E–G).

Thus, infection-induced resistance was associated with the generation of a heterogeneous B cell response and elevated anti-parasite IgG production, suggesting potential roles for atypical and/or regulatory B cells in promoting resistance to ECM.

#### Individual and Transitory Manipulation of Antibody and Atypical B Cell Populations Does Not Alter Resistance or Susceptibility to ECM

**Figure 5:**
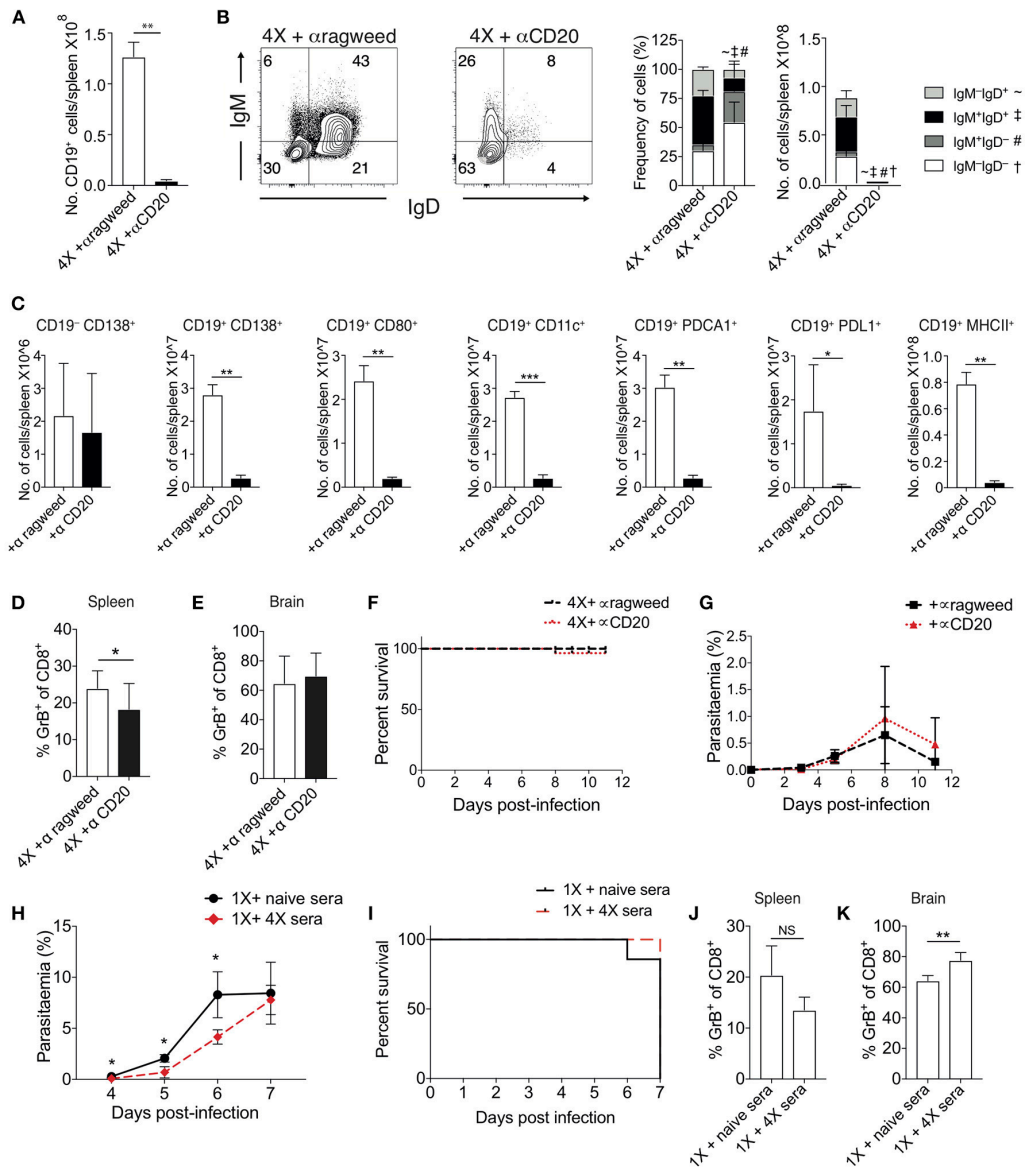
Ablation of B cell populations or transitory boosting of anti-parasite antibody does not alter dynamics of ECM in 1X or 4X infected mice. **(A–G)** C57BL/6 mice were injected (i.p) one day prior to 4X infection and on days 2, 5, 8, 11 of infection, with either (250 μg) anti-CD20 mAb or (250 μg) control anti-ragweed mAb. **(A)** Total numbers of splenic CD19^+^ B cells. **(B)** Left, Representative flow cytometric plots (gated on live CD45^+^CD3^−^CD19^+^ cells), right, the frequencies of non-switched (IgM^+^IgD^+^ and IgM^−^IgD^+^) and switched (IgM^+^IgD^−^, and IgM^−^IgD^−^) splenic CD19^+^ B cells. **(C)** Total numbers of splenic CD19^−^ CD138^+^ plasma B cells and splenic CD19^+^ B cells expressing CD138, CD80, CD11c, PDCA1, PDL-1, and MHC II in 4X infected mice that received anti-CD20 mAb or anti-ragweed mAb. **(D,E)** The frequencies of Granzyme B expressing CD8^+^ T cells in the **(D)** spleen and **(E)** brain in 4X infected mice that received anti-CD20 mAb or anti-ragweed mAb. **(F)** Survival of 4X infected mice given anti-CD20mAb or control mAb. **(G)** Parasitaemia (% of pRBCs) of 4X infected mice given anti-CD20mAb or control mAb. **(H–K)** C57BL/6 mice received 500 μl of heat-inactivated plasma (obtained from 4X infected mice or, as a control, from age-matched naïve mice) prior to and during 1X infection (4 i.p. injections between day −1 and day 5 of infection). **(H)** Peripheral parasitaemia (% of pRBCs) in 4X infected mice that received plasma from 4X or uninfected mice **(I)** Kinetics of ECM development shown as percentage survival of 1X infected mice that received plasma from 4X or uninfected mice. **(J,K)** The frequencies of granzyme B expressing CD8^+^ T cells in the **(J)** spleen and **(K)** brain in 1X infected mice that received plasma from 4X or naïve mice. **(A–K)** Results are the mean ± SD of the group. **(A–C)** *N* = 3–4 per group, representative of two independent experiments. **(D,E)** *N* = 7–8 per group, pooled from two independent experiments. **(F, G)** *N* = 10–13 per group, pooled from two independent experiments. **(H, I)** *N* = 6–7 per group, pooled from two independent experiments. **(J,K)** *N* = 4, representative of two independent experiments. Statistical analyses were performed with by Student's *t*-test, with Welch's correction or Mann-Whitney test depending on normality of data (^*^p ≤ 0.05, ^**^p ≤ 0.01 ^***^p ≤ 0.001 and *p* < 0.05 denoted by #, ~, ‡ for indicated groups).

To examine the importance of atypical B cell populations in infection-induced resistance to ECM, we treated mice immediately prior to and during 4X infection with anti-CD20mAb (to enable pan-B cell depletion). This approach was necessary due to the spectrum of cell populations that express atypical B cell markers, precluding the use of other specific antibody-targeting approaches. Anti-CD20 mAb depleted nearly all CD19^+^ B cells in 4X infected mice (Figure 5A); including the majority of class switched CD19^+^ B cells (Figure 5B). However, as previously reported (39), treatment had less of an effect on CD19^−^CD138^+^ plasma cells than on putative regulatory and antigen-presenting B cells (including CD19^+^PD-L1^+^, CD19^+^CD11c^+^, CD19^+^PDCA-1^+^, and CD19^+^MHC-II^+^ B cells), CD19^+^CD138^+^ plasmablasts and CD19^+^CD80^+^ activated / memory cells (Figure 5C). Nevertheless, despite the depletion of the majority of the B cell compartment, anti-CD20 mAb treatment did not increase the activation or function of splenic or intracerebral CD8^+^ T cells during 4th infection (Figures 5D,E), with granzyme B expression by CD8^+^ T cells in anti-CD20mAb and control Ab treated 4X infected mice being significantly lower than in 1X infection (Figure S4). Consequently, anti-CD20 mAb administration did not alter the resistance of 4X infected mice to ECM (Figures 5F,G). In agreement with this, blockade of IL-10 activity, a major mechanism of Breg suppression (38), did not reverse the resistance of 4X infected mice to ECM (Figure S4). This is despite the fact that IL-10 plasma levels trended higher in 4X infected mice compared with 1X infected mice (Figure S4), and adoptive transfer of IL-10 producing Bregs from repeatedly infected mice has previously been shown to promote resistance to ECM in primary infected mice (40).

The apparent redundancy of infection-induced atypical B cells (and other B cell populations) in actively mediating infection-induced resistance to ECM during the 4X infection suggested that circulating anti-parasite antibody (which was maintained at high level in pre-4X infected mice; Figures 4E,F), may be sufficient to confer protection against ECM. To investigate whether this was the case, we performed a passive transfer experiment by transferring plasma from 4X infected mice containing high titres of anti-parasite IgG (defined in Figures 4E,F) into mice during the course of 1X infection. Despite significantly lowering peripheral parasite levels during the early phases of 1X infection, passive transfer of plasma obtained from repeatedly infected mice (through 4 separate injections during the course of infection) failed to protect 1X infected mice from ECM (Figures 5H,I), or reduce CD8^+^ T cell activation within the spleen or brain (Figures 5J,K). Consequently, high levels of anti-parasite antibody did not appear to be sufficient to confer protection against ECM.

#### Mice Unable to Produce Secreted Antibody Fail to Acquire Infection-Induced Resistance to ECM

**Figure 6:**
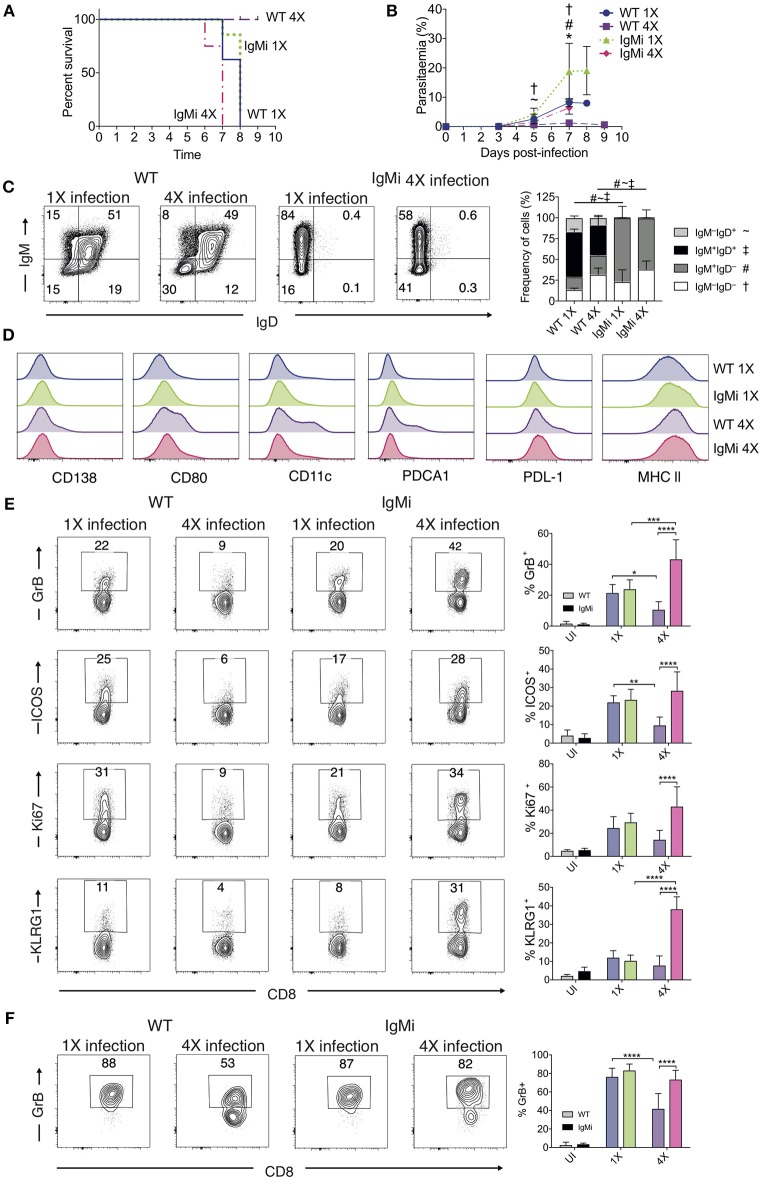
IgMi mice do not develop infection-induced resistance to ECM. **(A–F)** IgMi mice and WT littermate controls were infected with PbA (10^4^ pRBCs i.v.) or left uninfected. Mice were treated (i.p.) with chloroquine and artesunate from day 5 or 6 post each infection, and re-infections were performed after a minimum interval of 30 days following cessation of drug treatment. **(A)** Kinetics of ECM development, shown as percentage of survival of mice. **(B)** Peripheral parasitaemia (% of pRBCs). **(C)** Left, Representative flow cytometric plots (gated on live CD45^+^CD3^−^CD19^+^ cells), right, the frequencies of non-switched (IgM^+^IgD^+^ and IgM^−^IgD^+^) and switched (IgM^+^IgD^−^, and IgM^−^IgD^−^) splenic CD19^+^ B cells. **(D)** Expression of CD138, CD80, CD11c, PDCA1, PDL-1 and MHC II by CD19^+^ splenic B cells from 1X infected and 4X infected WT and IgMi mice. **(E,F)** Activation phenotype of **(E)** splenic and **(F)** brain accumulating CD8^+^ T cells. **(B,C,E,F)** Results are the mean ± SD of the group. **(A,B)** *N* = 7–8 per group, pooled from two independent experiments. **(C)** *N* = 3–4 per group, representative of two independent experiments. **(E,F)** *N* = 5–8 per group, pooled from two independent experiments. **(B,E,F)** Statistical analyses were performed with Kruskal-Wallis test with Dunn's multiple comparisons test or with one-way ANOVA with Tukey's test depending on normality of data (^*^p ≤ 0.05, ^**^p ≤ 0.01, ^***^p ≤ 0.001, ^****^p ≤ 0.0001, and *p* < 0.05 denoted as follows ^*^, WT 1X v IgMi 1X, ~, WT 4X v IgMi 1X, #, WT 4X v IgMi 4X, †, IgMi 1X v IgMi 4X). **(C)** Statistical analysis by Student's t-test, with Welch's correction for indicated groups (*p* < 0.05 denoted by #, ~, ‡ for the indicated groups).

The above results suggested that anti-parasite Ab may not be sufficient for active protection against ECM when given acutely during a primary infection. Antibody can directly and indirectly coordinate many events within the immune system, potentially influencing long-term immunity and controlling the nature of immune responses during challenge infections (41–44). As such, we reasoned that antibody may need to be produced and maintained during the initial rounds of PbA infection to condition (or educate) the immune system in repeatedly infected mice, eventually promoting resistance to ECM in 4X infected mice. To investigate if this hypothesis was correct, we used IgMi mice, which contain B cells that are able to produce membrane IgM but are unable to make secreted antibody or undergo class switching (21, 22), As expected, no anti- *Pb*MSP1_19_ or total anti-PbA IgG was detected in the plasma of 4X infected IgMi mice. Importantly, and in contrast to littermate WT mice, 4X infected IgMi mice failed to acquire complete or even partial resistance to ECM, with 100% (8/8) of mice developing accelerated late-stage ECM between days 6 and 7 p.i. (Figure 6A). Parasite control was reduced but not completely abrogated in 4X infected IgMi mice compared with 1X infected IgMi mice and 4X infected WT mice (Figure 6B).

As expected, essentially all CD19^+^ B cells in IgMi mice expressed IgM, but not IgD, during 1X infection and this expression pattern did not change during 4X infection (Figure 6C). Interestingly, although redundant for active resistance during 4X infection in WT mice, the development of atypical and/or regulatory CD11c^+^, PDCA-1^+^ and PDL-1^+^ B cell populations, as well as CD138^+^ and CD80^+^ populations, were abrogated in 4X IgMi mice (Figure 6D), indicating that infection-induced development of atypical B cell populations during malaria was dependent upon secreted antibody.

Splenic CD8^+^ T cell responses were significantly increased in 4X IgMi mice compared with 4X infected WT mice, as demonstrated by the significantly increased frequencies of CD8^+^ T cells expressing granzyme B, ICOS, Ki67, and KLRG1 in 4X infected IgMi mice compared with 4X infected WT mice (Figure 6E). Moreover, the frequencies of CD8^+^ T cells expressing granzyme B were higher in the brains of 4X infected IgMi mice compared with 4X WT mice (Figure 6F). Interestingly, despite lower peripheral parasitaemia, the expression of granzyme B and KLRG1 by splenic CD8^+^ T cells was enhanced rather than decreased in 4X compared with 1X infected IgMi mice, suggesting that that CD8^+^ T cell activation during repeated PbA infections was not directly proportional to peripheral parasite burden (Figures 6B,E). In contrast, the activation of splenic CD4^+^ T cell responses were unaltered in 4X IgMi mice compared with corresponding 4X infected WT mice (Figure S5).

Thus, collectively, our data suggest that secreted antibody is required during the repeated rounds of infection to modulate the B cell compartment and suppress pathogenic CD8^+^ T cell responses to establish infection-induced resistance to ECM.

## Discussion

In this study, we have shown that multiple rounds of PbA parasite exposure lead to the incremental development of ECM resistance in otherwise susceptible C57BL/6 background mice. Our results, therefore, appear to recapitulate the development of naturally acquired resistance to CM in humans in malarial endemic regions (4). Although protection against ECM was associated with the gradual control of peripheral parasite burdens and reduced parasite accumulation within the brain, sterile immunity was not achieved.

In this model system infection-induced protection against ECM appeared to depend upon the inhibition of memory CD8^+^ T cell reactivation, rather than a generalized dampening of inflammation. Indeed, splenic CD4^+^ T cell responses and plasma pro-inflammatory cytokine levels were comparable or increased in 4X infected mice compared with 1X infected mice. Granzyme B expression by CD8^+^ T cells was reduced in 4X infected mice compared with 1X infected mice but IFN-γ production was not significantly affected by the number of infections. It has been shown that IL-12/IL-18 and IL-15 differentially control IFN-γ production and granzyme B expression by reactivating memory CD8^+^ T cells, respectively (45). Consequently, it is foreseeable that modified memory CD8^+^ T cell reactivation during 4X infection was caused by alterations in the levels of IL-15. However, it is possible that lowered peripheral parasitaemia in 4X infected mice also contributed to alterations in CD8^+^ T cell reactivation.

It is probable that the inhibition of CD8^+^ T cell pathogenic responses in the brain of 4X infected mice was caused by the modulation of CD8^+^ T cell immune activation within the spleen, leading to reduced migration of less-pathogenic CD8^+^ T cells to the brain in 4X infected mice. Nevertheless, our transcriptomics analyses also suggest that many of the tissue signals, such as endothelial cell cross presentation of parasite antigen, which are necessary for CD8^+^ T cells to arrest, mediate damage to the blood brain barrier and provoke ECM (32, 46, 47), were also down regulated in the brains of 4X infected mice. This was likely, in part, due to the fact that parasite accumulation appeared, through qualitative assessment, to be reduced / prevented in brains of 4X compared with 1X infected mice. Indeed, it has previously been shown that pathogenic CD8^+^ T cell activation in the brain during *PbA* infection depends upon a threshold level of parasite accumulation and parasitic antigen in the brain (48). Interestingly, our transcriptomics analyses suggests that infection-induced dampening of intracerebral immune responses and resistance to ECM mirrors processes that specify genetic resistance to ECM, and those associated with avirulent parasite infections (49–51). The datasets generated in this study will be useful resources to identify other gene candidates that control resistance to ECM during *Pb*A infection.

In terms of the infection-induced processes that provided protection against ECM, we have shown the critical non-redundant role of antibody. IgMi mice, which were unable to produce secreted antibody or undergo class switching (21, 22), developed hyperactive splenic and intracerebral CD8^+^ T cell responses during 4X infection and consequently failed to acquire infection-induced resistance to ECM. Antibody responses to the immunodominant antigen MSP-1_19_ did not correlate with resistance to ECM; however, total anti-parasite Ab responses developed more gradually over repeated infections and were eventually maintained, at least short term, between 3rd and 4th infections. These observations are in agreement with previous studies showing that anti- *Pb*MSP1 antibody titres did not correspond with vaccine induced protection against PbA (30). Also, they are consistent with studies in human malaria, showing that exposure-induced protection against *P. falciparum* is associated with the gradual accumulation of B cells with different specificities (12, 52, 53). The role of maintained antibody in infection-induced protection to ECM is supported by seroepidemiological studies in humans, where the level of anti-*P. falciparum* antibody prior to the malaria season, but not after the malaria season, correlated with protection against clinical disease (12, 52).

The fact that IgMi mice failed to develop resistance to ECM but we were unable to protect 1X infected mice by passive transfer of plasma from 4X infected mice may suggest that we failed to transfer enough antibody in our experiments to promote protection. Whilst we cannot fully discount this possibility, passive transfer of plasma from 4X infected mice did lead to significant reductions in parasite levels during the course of 1X infection. Moreover, we transferred higher amounts of antibody (four injections of 500 μl of plasma with Ab end point titer >1 × 10^6^ during the course of infection) than has been used to significantly reduce parasite burdens and protect mice from other malarial species, such as *P. yoelii* and *P. chabaudi* (31, 54). The inability of passively transferred anti-parasite Ab to provide protection against *P. berghei* infection has previously been described (54) and, to date, we are unaware of any other study where passive transfer of antisera has promoted resistance against ECM in primary PbA infected mice. Thus, our data argue that repeated PbA infection promotes generation and maintenance of anti-parasite antibodies that establish changes in the immune system over the course of repeat infections that provide protection against ECM, and which cannot be easily recapitulated by acute administration of high titres of antibody to primary infected mice.

There are various potential ways through which antibody could educate the immune system during the course of repetitive *Plasmodium* infections to inhibit CD8^+^ T cell activity and promote resistance to ECM. Antibody, in the form of immune complexes and via triggering Fc receptors, or through activating complement, can significantly modulate APC activation, cytokine production and antigen presentation (41–44). There is accumulating evidence that memory CD8^+^ T cell reactivation can be substantially influenced by the repertoire, duration and level of cytokine production, co-stimulation, and nature of antigen presentation from different APC populations (45, 55–57). For example, in viral infections antibody-antigen immune complexes can extend the duration of antigen presentation post-clearance of infection, which although not affecting the ultimate magnitude of the maintained memory CD8^+^ T cell population, significantly influences memory CD8^+^ T cell reactivation (41). Notably, modulation of innate cell phenotype and function can persist post-clearance of *Plasmodium* infection (58), and the duration of these changes have been linked to the maintenance of anti-parasite antibody (59). It has also been reported that the CD11c^+^ DC population is altered in mice repeatedly infected with PbA and that this contributes to resistance to ECM (60). Thus, antibody may extend infection-induced alterations in innate cell activity and antigen presentation following clearance of infection, as well as influencing APC reactivity in the acute period during challenge infections, ultimately affecting memory CD8^+^ T cell reactivation. Although we focussed on anti-parasite IgG in this study, it has been shown anti-malarial memory B cell populations maintain the capacity for IgM production (61). IgM through binding to TOSO on B cells has been shown to indirectly regulate CD8^+^ T cell activation during PbA infection (62). Thus, secreted IgM and IgG isotypes may play a role in regulating memory CD8^+^ T cell reactivation during repeated *Plasmodium* spp. infection. Defining precisely how antibody, and importantly gradual acquisition of different specificities of anti-parasite Ab, influence the immune environment to repress pathogenic CD8^+^ T cell responses during 4X PbA infection to promote resistance to ECM should be the subject of detailed investigations in further studies. For example, the role of the Fcgamma RIIb receptor, which is expressed on CD8^+^ T cells and which influences memory CD8^+^ T cell activation during Listeria monocytogenes infection (63), could be examined by studying whether Fcgamma RIIb deficient mice develop infection-induced resistance to ECM.

Whilst we believe antibody was key for protection in our infection-drug cure model, it was of interest that infection-induced protection against ECM also corresponded with the formation of atypical B cell populations in the spleen, and that development of these atypical B cell populations was abrogated in repeatedly infected IgMi mice. The function of atypical B cell populations during malaria, which have principally be defined as memory B cells (12–15), but in our model could also include non-conforming plasmablasts and plasma cells (16, 33, 34), is still unclear. Very recently it has been shown that atypical B cells are short-lived activated B cells during malaria that form as part of a natural ongoing B cell response (64). Evidence suggests atypical B cells may be protective through anti-parasite Ab production (15) or via immunoregulatory activity, suppressing damaging inflammation (14, 17–19), or they are dysfunctional, impeding generation of long-lived humoral immunity or contributing to autoimmunity (12–16, 19). We found that transitory depletion of the majority of atypical B cells as well as CD19^+^IgM^+^IgD^+^ cells [which include classical regulatory B cells (38)] immediately prior to and during 4X infection by anti-CD20 mAb administration failed to reverse infection-induced resistance to ECM. This showed that atypical B cells and regulatory B cell populations (as well as other classical B cell populations) were not necessary for active protection to ECM during the on-going 4X infection, at least when the duration between 3 and 4X infection was relatively short. This result contrasts with the previous observation that adoptive transfer of IL-10 expressing Bregs from ECM resistant multiply infected mice promotes resistance to ECM in primary infected mice (40). Whilst the reason for our conflicting data is unclear it is probable that although high numbers of IL-10 expressing Bregs can be sufficient for protection against ECM following adoptive transfer, they are not necessary for physiological active protection in the context of a full working memory immune compartment. In agreement with this, we found that IL-10R blockade, although slightly improving parasite control, did not affect the resistance of 4X infected WT mice to ECM. Nevertheless, we cannot discount the possibility that the atypical B cell populations, in concert with antibody, may play a role in gradually modulating the immune system during repetitive infections, eventually conditioning an immunological state that provides resistance to ECM in 4X infected mice. Indeed, B cells can undertake APC functions and can directly tolerise CD8^+^ T cells (65). The overall role of B cells in establishing anti-disease immunity could be examined in the future using uMT mice.

In summary, in this model of repeated parasite exposure we have shown that infection-induced resistance to ECM was associated with the repression of memory CD8^+^ T cell reactivation during 4X infection. Resistance to ECM and inhibition of pathogenic memory CD8^+^ T cell responses was ablated in mice unable to produce secreted antibody but protection against ECM could not be recapitulated solely by the transfer of high titer anti-parasite antibody prior to primary infection, and protection during the fourth infection was not reversed by active depletion of the majority of the B cell compartment. Thus, our results suggest that anti-parasite Ab may gradually orchestrate and maintain changes in the immunological environment that eventually attenuate splenic memory CD8^+^ T cell reactivation in repeatedly infected mice. Although the contribution of CD8^+^ T cells in the pathogenesis of HCM remains controversial, there is some evidence that activated CD8^+^ T cells may play a role in severe malarial disease (66, 67). Consequently, our results provide insight into the mechanisms of protection to ECM and may have implications for studying the basis of infection-induced resistance to severe malarial disease and specifically cerebral malaria in humans.

### Ethics

Animal work was approved following local ethical review by the Universities of Manchester and Oxford Animal Procedures and Ethics Committees and was performed in strict accordance with the U. K Home Office Animals (Scientific Procedures) Act 1986 (approved H.O Project Licenses 70/7293, 30/2889, and P8829D3B4).

## Data Availability

The microarray datasets reported in this paper have been deposited in the ArrayExpress database (accession number E-MTAB-5513).

## Author Contributions

TS and KC: conceptualization; TS, CI, PS, DP, SD, and KC: methodology; TS, CI, DP, PS, and AV-M: investigation; LZ: formal Analysis; TS and KC: writing—Original Draft; TS and KC: writing—review and editing; TS and KC: funding acquisition; SD and KE: resources; KC: supervision; TS: visualization.

### Conflict of Interest Statement

The authors declare that the research was conducted in the absence of any commercial or financial relationships that could be construed as a potential conflict of interest.

## Supplementary Material

Figure S1 **(A)** Peripheral parasitaemia (% of pRBCs) ± SD in 3X and 4X infection groups from day 8 post infection (*n* = 2–10 per time point). **(B)** Representative H & E stained brain sections demonstrating presence and absence of hemorrhage, packed vessels and oedema in 1X and 4X infected mice on day 8 post infection. **(C)** Representative immunofluorescence stained cortical brain sections demonstrating presence and absence of pRBCs (green) in the CD31^+^ cerebrovascular (red) of 1 and 4X infected mice on day 8 post infection. Nuclei were counterstained with dapi (blue).Scale bar 25 μm.

Figure S2 **(A–D)** Perfused whole brains were removed from 4X infected and age-matched 1X infected C57BL/6 mice on day 8 p.i. (when 1X developed ECM), and age-matched nal¨ve mice, for microarray analysis. **(A)** K-means and hierarchical clustering of differentially expressed genes in 4X mice vs. uninfected mice and 1X mice vs. uninfected mice. Each probe-set expression level was normalized to the naïve average. **(B)** Gene ontology analysis identifying enriched biological processes within each gene cluster, identified within DAVID bioinformatics database. **(C)** Full size defense response and **(D)** regulation of apoptosis gene ontology pathways differentially expressed in brains of 1X and 4X infected mice. *N* = 6 per group. Results are generated from the pooled array data from brains taken from two independent experiments.

Figure S3 **(A,B)** Perfused whole brains were removed from 4X infected and age-matched 1X infected C57BL/6 mice on day 8 p.i. (when 1X developed ECM), for microarray analysis. Ingenuity analysis identified **(A)** IL-6- and **(B)** IFN-γ-controlled gene networks as two major pro-inflammatory gene networks downregulated in the brains of 4X infected mice compared with 1X infected mice (green color represents down-regulated gene expression and red color represents up-regulated gene expression). **(C)** Nanostring validation of expression of selected genes in whole brains of 1 and 4X infected mice on day 8 of infection (presented as fold change in expression compared with nal¨ve brains). **(A,B)** *N* = 6 per group. Results are generated from the pooled array data from brains taken from two independent experiments. **(C)** *N* = 5 per group, from two pooled experiments. Statistical analysis by Student's *t*-test, with Welch's correction (^*^*p* ≤ 0.05, ^**^*p* ≤ 0.01, ^****^*p* ≤ 0.0001).

Figure S4 **(A,B)** C57BL/6 mice were injected (i.p) one day prior to 4X infection and on days 2, 5, 8, 11 of infection, with either (250 μg) anti-CD20 mAb or (250 μg) control anti-ragweed mAb. Frequencies of granzyme B expressing CD8^+^ T cells in **(A)** the spleen and **(B)** the brain on day 8 post infection of age matched nal¨ve, 1X infected and 4X infected mice, that received anti-CD20 mAb or anti-ragweed mAb. **(C)** Cytokine bead array of plasma cytokine IL-10 levels in 4X, 1X infected mice and aged matched uninfected C57BL/6 mice. **(D)** C57BL/6 mice were injected (i.p) one day prior to the 4X infection and on every other day of 4X infection with anti-IL-10R mAb or PBS. Kinetics of ECM development shown as percentage survival of mice. **(A–C)** Results are the mean ± SD of the group. **(A,B)** *N* = 4–8 per group, pooled from two independent experiments. **(C)** *N* = 4–7 per group, pooled from two independent experiments. **(D)** *N* = 9 per group, pooled from two independent experiments. Statistical analyses were performed with Kruskal-Wallis test with Dunn's multiple comparisons test (^*^*p* ≤ 0.05, ^**^*p* ≤ 0.01 and ^***^*p* ≤ 0.001).

Figure S5 IgMi mice and WT littermate controls were infected with PbA (10^4^ pRBCs i.v.) or left uninfected. Mice were treated (i.p.) with chloroquine and artesunate from day 5 or 6 post each infection, and re-infections were performed after a minimum interval of 30 days following cessation of drug treatment. Activation phenotype of splenic CD4^+^ T cells in the different groups of IgMi and WT littermate mice. *N* = 2–4 per group, representative of two independent experiments. Statistical analyses were performed with Kruskal-Wallis test with Dunn's multiple comparisons test (^*^*p* ≤ 0.05).

Supplementary Table 1 C57BL/6 mice were infected with PbA (104 pRBCs i.v.) or left uninfected. Mice were treated (i.p.) with chloroquine and artesunate as shown in Figure 1A, and re-infections were performed after a minimum interval of 30 days following cessation of drug treatment. Table shows the day post infection, number of mice, mean peripheral parasitaemia (% of pRBCs) ± SD in different infection groups. Results are pooled from two experiments for the 1X, 2X, and 3X infection and from 3 experiments for the 4X infection.

Supplementary Table 2 List of differentially expressed genes included within Figure 2 and Figure S2.

Supplementary Table 3 Genes in Supplementary Table 1 filtered to identify genes differentially expressed between 4X and 1X brains.
