# Supplementary figures and images for: Infection-Induced Resistance to Experimental Cerebral Malaria Is Dependent Upon Secreted Antibody-Mediated Inhibition of Pathogenic CD8+ T Cell Responses
Source: Front Immunol. 2019 Feb 19;10:248. doi: 10.3389/fimmu.2019.00248 (PMC6394254; doi:10.3389/fimmu.2019.00248)

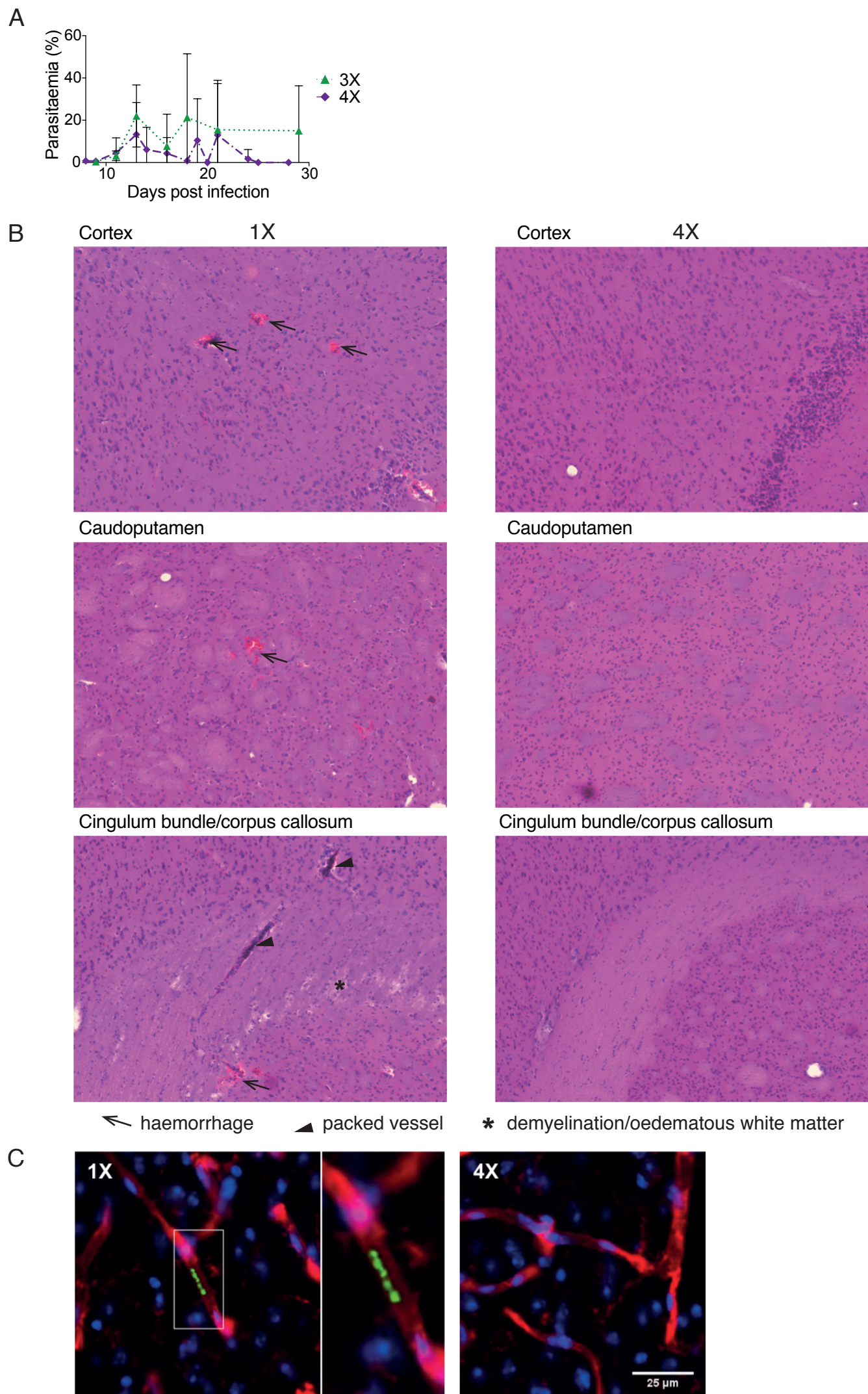

Figure S1

Supplement: Figure S1 — (A) Peripheral parasitaemia (% of pRBCs) ± SD in 3X and 4X infection groups from day 8 post infection (n = 2–10 per time point). (B) Representative H & E stained brain sections demonstrating presence and absence of hemorrhage, packed vessels and oedema in 1X and 4X infected mice on day 8 post infection. (C) Representative immunofluorescence stained cortical brain sections demonstrating presence and absence of pRBCs (green) in the CD31+ cerebrovascular (red) of 1 and 4X infected mice on day 8 post infection. Nuclei were counterstained with dapi (blue).Scale bar 25 μm. [file Data_Sheet_1.PDF]

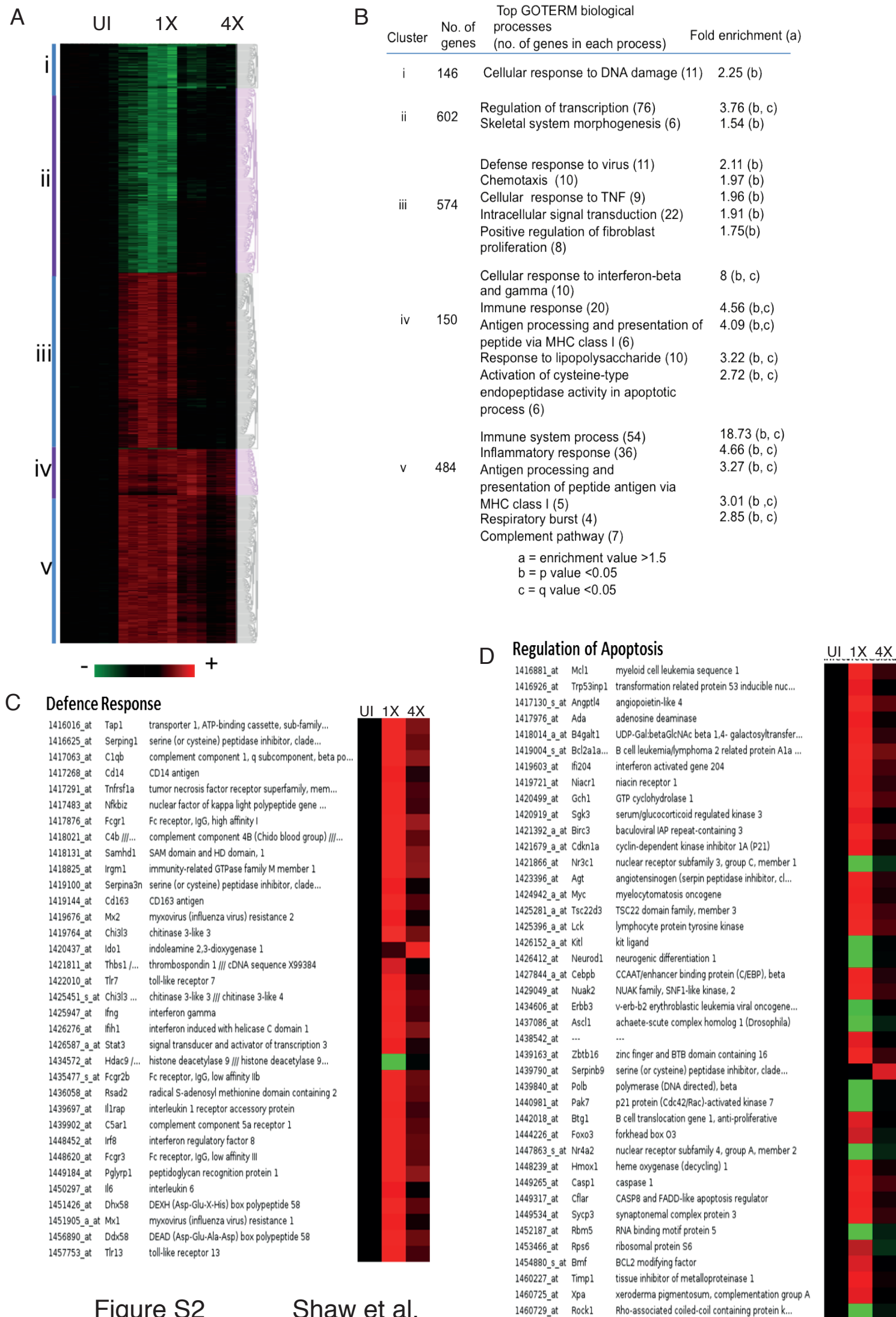

Figure S2

Shaw et al.

Supplement: Figure S2 — (A–D) Perfused whole brains were removed from 4X infected and age-matched 1X infected C57BL/6 mice on day 8 p.i. (when 1X developed ECM), and age-matched nal¨ve mice, for microarray analysis. (A) K-means and hierarchical clustering of differentially expressed genes in 4X mice vs. uninfected mice and 1X mice vs. uninfected mice. Each probe-set expression level was normalized to the naïve average. (B) Gene ontology analysis identifying enriched biological processes within each gene cluster, identified within DAVID bioinformatics database. (C) Full size defense response and (D) regulation of apoptosis gene ontology pathways differentially expressed in brains of 1X and 4X infected mice. N = 6 per group. Results are generated from the pooled array data from brains taken from two independent experiments. [file Data_Sheet_2.PDF]

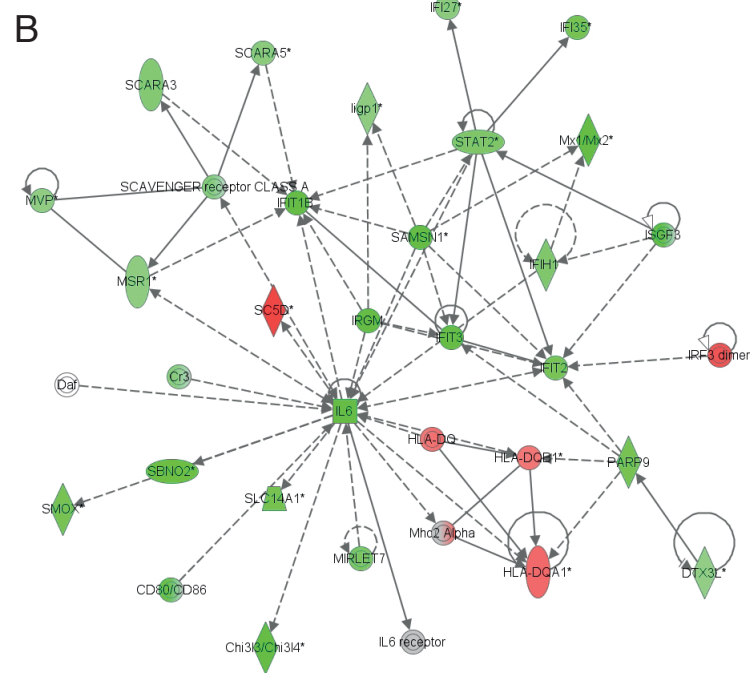

© 2000-2015 QIAGEN. All rights reserved.

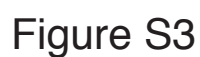

Supplement: Figure S3 — (A,B) Perfused whole brains were removed from 4X infected and age-matched 1X infected C57BL/6 mice on day 8 p.i. (when 1X developed ECM), for microarray analysis. Ingenuity analysis identified (A) IL-6- and (B) IFN-γ-controlled gene networks as two major pro-inflammatory gene networks downregulated in the brains of 4X infected mice compared with 1X infected mice (green color represents down-regulated gene expression and red color represents up-regulated gene expression). (C) Nanostring validation of expression of selected genes in whole brains of 1 and 4X infected mice on day 8 of infection (presented as fold change in expression compared with nal¨ve brains). (A,B) N = 6 per group. Results are generated from the pooled array data from brains taken from two independent experiments. (C) N = 5 per group, from two pooled experiments. Statistical analysis by Student's t-test, with Welch's correction (*p ≤ 0.05, **p ≤ 0.01, ****p ≤ 0.0001). [file Data_Sheet_3.PDF]

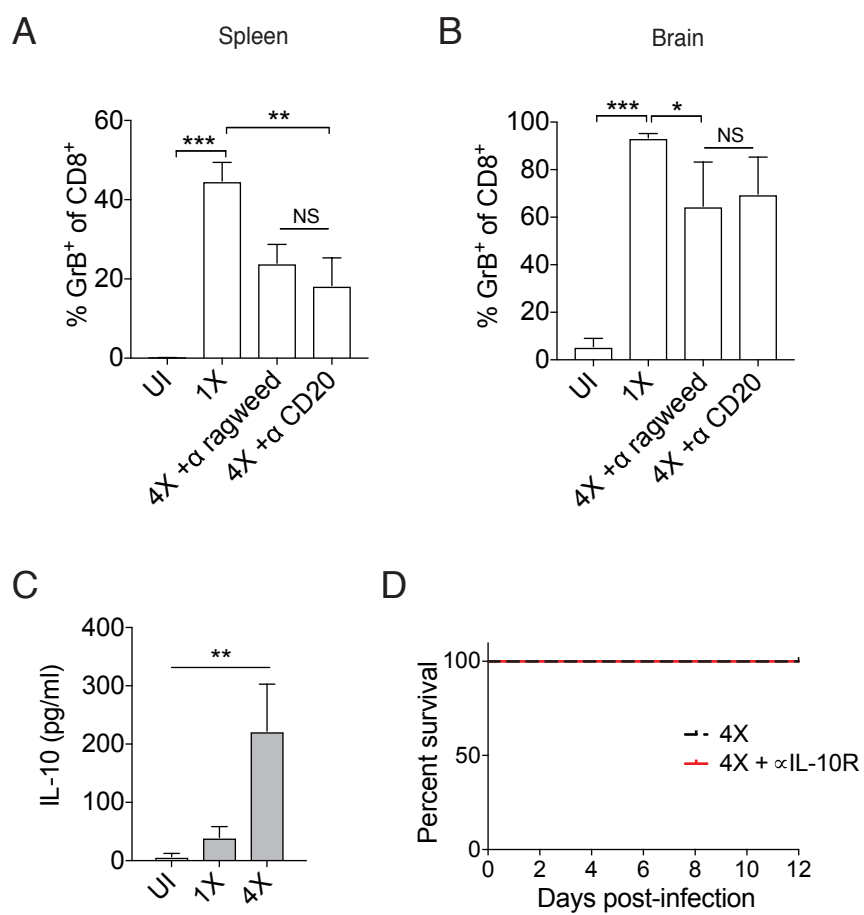

Figure S4

Shaw et al.

Supplement: Figure S4 — (A,B) C57BL/6 mice were injected (i.p) one day prior to 4X infection and on days 2, 5, 8, 11 of infection, with either (250 μg) anti-CD20 mAb or (250 μg) control anti-ragweed mAb. Frequencies of granzyme B expressing CD8+ T cells in (A) the spleen and (B) the brain on day 8 post infection of age matched nal¨ve, 1X infected and 4X infected mice, that received anti-CD20 mAb or anti-ragweed mAb. (C) Cytokine bead array of plasma cytokine IL-10 levels in 4X, 1X infected mice and aged matched uninfected C57BL/6 mice. (D) C57BL/6 mice were injected (i.p) one day prior to the 4X infection and on every other day of 4X infection with anti-IL-10R mAb or PBS. Kinetics of ECM development shown as percentage survival of mice. (A–C) Results are the mean ± SD of the group. (A,B) N = 4–8 per group, pooled from two independent experiments. (C) N = 4–7 per group, pooled from two independent experiments. (D) N = 9 per group, pooled from two independent experiments. Statistical analyses were performed with Kruskal-Wallis test with Dunn's multiple comparisons test (*p ≤ 0.05, **p ≤ 0.01 and ***p ≤ 0.001). [file Data_Sheet_4.PDF]

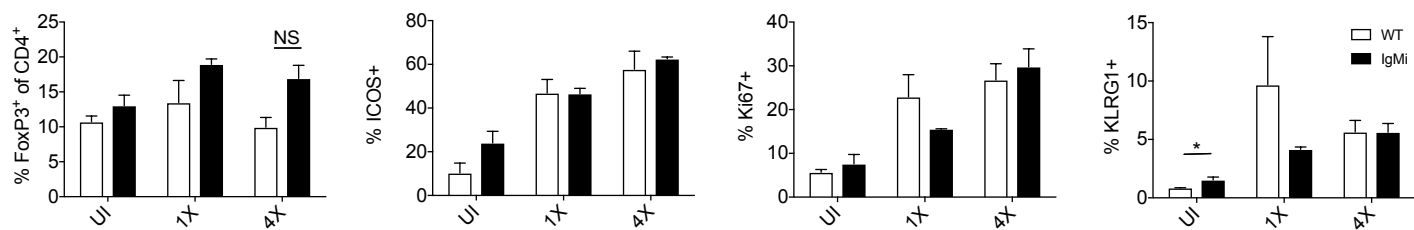

Figure S5

Shaw et al.

Supplement: Figure S5 — IgMi mice and WT littermate controls were infected with PbA (104 pRBCs i.v.) or left uninfected. Mice were treated (i.p.) with chloroquine and artesunate from day 5 or 6 post each infection, and re-infections were performed after a minimum interval of 30 days following cessation of drug treatment. Activation phenotype of splenic CD4+ T cells in the different groups of IgMi and WT littermate mice. N = 2–4 per group, representative of two independent experiments. Statistical analyses were performed with Kruskal-Wallis test with Dunn's multiple comparisons test (*p ≤ 0.05). [file Data_Sheet_5.PDF]
