# Supplementary material for: Infection-Induced Resistance to Experimental Cerebral Malaria Is Dependent Upon Secreted Antibody-Mediated Inhibition of Pathogenic CD8+ T Cell Responses
Source: Front Immunol. 2019 Feb 19;10:248. doi: 10.3389/fimmu.2019.00248 (PMC6394254; doi:10.3389/fimmu.2019.00248)
Supplement: Supplementary Table 1 — C57BL/6 mice were infected with PbA (104 pRBCs i.v.) or left uninfected. Mice were treated (i.p.) with chloroquine and artesunate as shown in Figure 1A, and re-infections were performed after a minimum interval of 30 days following cessation of drug treatment. Table shows the day post infection, number of mice, mean peripheral parasitaemia (% of pRBCs) ± SD in different infection groups. Results are pooled from two experiments for the 1X, 2X, and 3X infection and from 3 experiments for the 4X infection. [file Table_1.pdf]

Supplementary Table 1.

Infection no.	Day post infection	No. of mice	Mean parasitaemia	SD
1X	0	6	0.00	0.00
	3	6	0.02	0.03
	5	6	2.22	0.88
	7	9	9.85	3.32
2X	0	8	0.00	0.00
	3	9	0.14	0.08
	5	8	2.10	0.91
	6	7	3.15	1.03
	7	7	4.45	1.08
3X	0	5	0.00	0.00
	4	5	0.06	0.07
	5	5	0.41	0.26
	6	5	1.74	0.81
	7	9	2.76	0.96
	9	5	0.43	0.24
	11	3	3.26	2.30
	13	5	22.04	14.68
	16	4	7.79	15.21
	18	2	21.35	30.19
	21	2	15.50	21.92
	28	2	15.05	21.28
4X	0	7	0.00	0.00
	3	8	0.01	0.02
	5	8	0.17	0.14
	7	8	1.29	0.87
	8	7	0.80	0.67
	9	3	0.63	0.77
	11	7	4.62	7.16
	13	10	13.31	15.22
	14	4	6.09	10.56
	16	3	4.33	7.50
	18	3	0.81	1.40
	19	6	10.50	19.78
	20	3	0.00	0.00
	21	4	12.98	25.95
	24	6	1.81	4.43
	25	3	0.00	0.00
	28	6	0.00	0.00
